# Rehydration characteristics of milk protein concentrate powders monitored by electrical resistance tomography

**DOI:** 10.3168/jdsc.2021-0125

**Published:** 2021-09-13

**Authors:** K.S. Babu, J.K. Amamcharla

**Affiliations:** Department of Animal Sciences and Industry/Food Science Institute, Kansas State University, Manhattan 66506

## Abstract

Electrical resistance tomography (ERT) is a robust and low-cost method offering real-time visualization of processes. In this work, we developed an ERT-based method to characterize the rehydration behavior of milk protein concentrate (MPC) powders. Circular-type and linear configurations were used to achieve high resolution in the radial and axial directions, respectively. To evaluate the rehydration profile, MPC powders were reconstituted to 2.5% (wt/wt) total solids at room temperature, and the rehydration behavior of the MPC powders [MPC with 85% protein (MPC85) and milk protein isolate with 90% protein (MPI90)] was monitored for a dissolution time of 30 min using the ERT system. The MPC powders were characterized in terms of overall mean conductivity, area under the mean conductivity curve, slope at a dissolution time of 3 min, and the relative dissolution index. Additionally, the focus beam reflectance measurement (FBRM) was used as a reference method to follow rehydration characteristics. Particle count changes from the FBRM measurements showed that MPI90 had higher larger particle counts and more resistance to dispersing in water. As the dissolution time proceeded, mineral ions and proteins were released and consequently increased the overall conductivity, confirming the transfer of water into MPC particles. At lower protein contents, the particle dispersion rate was higher and an increase in overall mean conductivity was observed, indicating better powder dissolution. Both configurations were able to effectively monitor differences in the dissolution behavior of MPC powders. In the ERT circular configuration, MPC85 and MPI90 showed maximum conductivity of 0.201 ± 0.006 and 0.162 ± 0.001 mS/cm, respectively. In the linear probe configuration, MPC85 and MPI90 showed maximum conductivity of 0.161 ± 0.001 and 0.136 ± 0.001 mS/cm, respectively, suggesting increasingly inhibited water transfer as the protein content of the powder increased. In this study, we demonstrated the capability of ERT using the circular and linear probe configurations to offer, in addition to qualitative tomographic images, reliable quantitative data by which to characterize the dissolution behavior of high-protein dairy powders.

## Highlights

• We used electrical resistance tomography (ERT) to examine milk protein concentration (MPC) powder rehydration.
• ERT facilitated real-time and in-line monitoring of the MPC powder dissolution process.
• The rate of water diffusion was higher for low protein powders.

## Graphical Abstract

**Figure:**
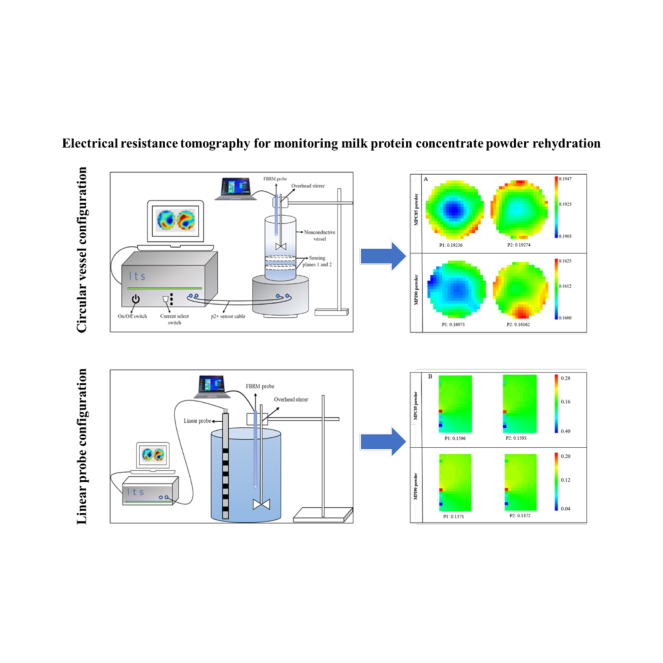
**Summary:** Milk protein concentrate (MPC) powders are dairy ingredients with protein contents ranging from 35 to 90% (wt/wt), and they are widely used in various food product formulations to improve their nutritional, functional, and sensory properties. However, it is a challenge for the end-user to incorporate MPC powders in a formulation because of their poor rehydration properties. This is related to the powder manufacturing process and subsequent storage. Therefore, there is a need to develop potential in-line techniques to monitor MPC powder rehydration. Methods available to monitor the rehydration of powders can either distinguish between multiple rehydration stages or measure total rehydration time based on specific phenomena related to rehydration. The protein and mineral release during rehydration of MPC powders can be followed by conductivity measurements. In this study, we assessed the rehydration behavior of MPC powders (MPC85 and MPI90) in real time using electrical resistance tomography measurements.

Their high protein and low lactose content combined with desirable functional and nutritional attributes make milk protein concentrate (**MPC**) powders an ideal ingredient in various dairy and food product formulations. However, impaired rates of hydration and overall loss of rehydration characteristics during storage are key challenges for end-users to incorporate the MPC powders in a formulation. The reconstitution of MPC powders generally consists of 4 phases: wetting of powder particles, sinking, dispersing, and complete dissolution in the aqueous phase (Crowley et al., 2016). Protein content, storage time and temperature, and dissolution temperature are some of the critical factors that influence the reconstitution of MPC powders (Fyfe et al., 2011; Hauser and Amamcharla, 2016b; Babu and Amamcharla, 2018a).

Crowley et al. (2016) summarized various in-line and off-line techniques currently available for monitoring of MPC powder rehydration characteristics. Some of the currently available techniques can distinguish between different stages of rehydration, whereas others only measure total rehydration based on particular events related to rehydration (e.g., mineral release, water penetration). Techniques such as focus beam reflectance measurement (**FBRM**) and ultrasound-based measurement can offer in-line capabilities, making them more relevant for monitoring MPC powder rehydration without requiring sampling for off-line analysis. Marabi et al. (2008) studied a model dairy-based powder containing skim milk powder and noted that electrical conductivity could be a useful tool for investigating the rehydration profile of powders. Ions, amino acids, and proteins primarily contribute to electrical conductivity in a milk system, whereas lactose and lipids are poor conductors of electricity (Therdthai and Zhou, 2001). Previously, Gianfrancesco et al. (2011) used electrical conductivity as a tool to evaluate rehydration profiles of proteins and reported that heat-denatured β-lactoglobulin powder and sodium caseinate had longer rehydration times (indicating slower rehydration) than native β-lactoglobulin powder, suggesting that electrical conductivity was adequately sensitive to detect changes in powder rehydration induced by structural modifications to proteins. Indeed, none of the previous studies using the conductivity probe have sufficient data for real-time visualization or physical modeling of the dissolution process. Thus, a more robust and sensitive technique is needed to follow the high-protein powder dissolution process.

In recent years, electrical resistance tomography (**ERT**) has been explored for many industrial processes because it is a non-intrusive technique and provides key advantages over other monitoring techniques. It is a low-cost technique, can be easily implemented in-line or off-line, and offers high-speed monitoring capability. Electrical resistance tomography can also provide qualitative tomographic images and reliable quantitative data during material distribution in various systems. The ERT system consists primarily of 3 units: an electrode array, a data-acquisition system, and an image reconstruction system. The electrode array that is mounted on the circular vessel or linear probe can generate an electrical field within the region of interest by applying excitation signals. Resultant signals are acquired using electrodes mounted on the periphery of the circular vessel or linear probe (Aw et al., 2014). An accurate and stable data-acquisition system is critical to an ERT system. Excitation signal generation, electrode status control, signal conditioning, and demodulation are critical to monitoring real-time conductivity changes. Subsequently, data are sent to a host computer for image reconstruction using a suitable reconstruction algorithm (Jamaludin et al., 2013; Aw et al., 2014).

The wide range of applications of ERT, including mixing operations and other processes, was recently reviewed by Bowler et al. (2020). Tan et al. (2016) examined 2-phase oil-water flow behavior in a horizontal pipe using a 16-electrode ERT system. The authors demonstrated the change in flow patterns of oil to water using ERT. The technique is also widely used to investigate many single-phase and 2-phase systems in stirred vessels (Naeeni and Pakzad, 2019). In dairy applications, Sharifi and Young (2011) used ERT to detect anomalies and defects in milk mixing or holding tanks (milk inhomogeneity, adulteration, cream separation, recognition of powder lumps or external objects, aeration, and vortex development). Keeping the various advantages of ERT in mind, the objective of this study was to develop a method to characterize the rehydration behavior of MPC powders using the ERT system in both the circular vessel and linear probe configurations and to compare the ERT-based method with the previously developed FBRM-based method as a reference method.

For this purpose, 2 lots of fresh MPC with 85% protein (**MPC85**) and milk protein isolate with 90% protein (**MPI90**) powders were procured from a commercial manufacturer (Idaho Milk Products) to develop and evaluate the ERT-based method. According to the certificate of analysis provided by the manufacturer, the composition of MPC85 powders used in this study was approximately 83% protein, 5% moisture, 6% ash, 4% lactose, and 1.2% fat by weight. The MPI90 powders used in this study contained around 88% protein, 4.5% moisture, 6% ash, 0.5% lactose, and 1.2% fat by weight.

In our experiments, we used the ITS p2+ system (Industrial Tomography Systems) in 2 configurations (circular vessel and linear probe configurations) to monitor the dissolution of MPC powders. Real-time tomographic data were gathered by the sensors during the dissolution process as they evolved, based on contrasts in the electrical properties during powder dissolution. Figure 1 shows the experimental setup for the circular (panel A) and linear (panel B) probe configurations, respectively, along with the FBRM (Particle Track E25, Mettler Toldeo) setup. We used FBRM as the reference method to follow rehydration characteristics (Hauser and Amamcharla, 2016b). From our preliminary optimization studies, a 2.5% (wt/wt) concentration was found to be optimal for characterizing powder dissolution using the ERT-based method. To evaluate the method, powders were reconstituted to 2.5% (wt/wt) total solids at room temperature, and the rehydration behavior of the powders were monitored for a dissolution time of 30 min. In the circular vessel configuration setup, 17.50 g of MPC powder was gradually added to 682.50 g of tap water. In the linear probe configuration, 50 g of MPC powder was gradually added to 1,950 g of tap water. A 4-bladed overhead stirrer (Caframo) was placed along with the FBRM probe for the circular and linear configurations. The overhead stirrer speed was set to 900 rpm for the duration of powder addition to reduce the time required for powder particle wetting. The powder was then added over a period of <1 min. Subsequently, the stirrer speed was reduced to 400 rpm for the rest of the experiment. The ERT system generated a cross-sectional image showing the electrical conductivity distribution of a process from measurements from the circular vessel or the linear probe. The p2+ system introduces a current between a pair of electrodes and measures the resultant voltage difference between the remaining electrode pairs according to the predefined measurement protocol. Briefly, 2 sensing planes were selected to set up the p2+ system as suggested by the manufacturer, and the numbers of electrodes in a single measurement sensing plane were 16 and 8 for the ERT circular vessel and linear probe, respectively. All electrodes were made of stainless steel 316L, and the conductivity range selected was 0.05 to 100 mS/cm. All measurements were done at an injection frequency and current of 9,600 Hz and 1.5 to 15 mA, respectively. The current and gains were calibrated, and the reference conductivity (σ_reference_) measurement was carried out with tap water as the reference liquid. The ERT system was set up to calculate the relative conductivity measurements (σ_sample_/σ_reference_) of the sample during dissolution of MPC, and the relative conductivity data during dissolution were acquired using ITS p2+ software (version 9; Industrial Tomography Systems). A linear back projection image reconstruction algorithm was used to reconstruct the tomograms during powder dissolution, and then powder rehydration behavior was characterized in terms of overall mean conductivity. The ERT data were exported from the p2+ software to Excel (Microsoft Corp.). Subsequently, overall mean conductivity was plotted against powder dissolution time. From this curve, the area under the overall mean conductivity curve (calculated using the trapezoidal rule), the slope at powder dissolution of 3 min, and the relative dissolution index were extracted to quantitatively characterize powder dissolution. The relative dissolution index was derived for the MPC powders according to a method described by Babu et al. (2018). The ANOVA and significance were indicated by *P* < 0.05, using the statistical software SAS (SAS Institute Inc.).

**Figure 1:**
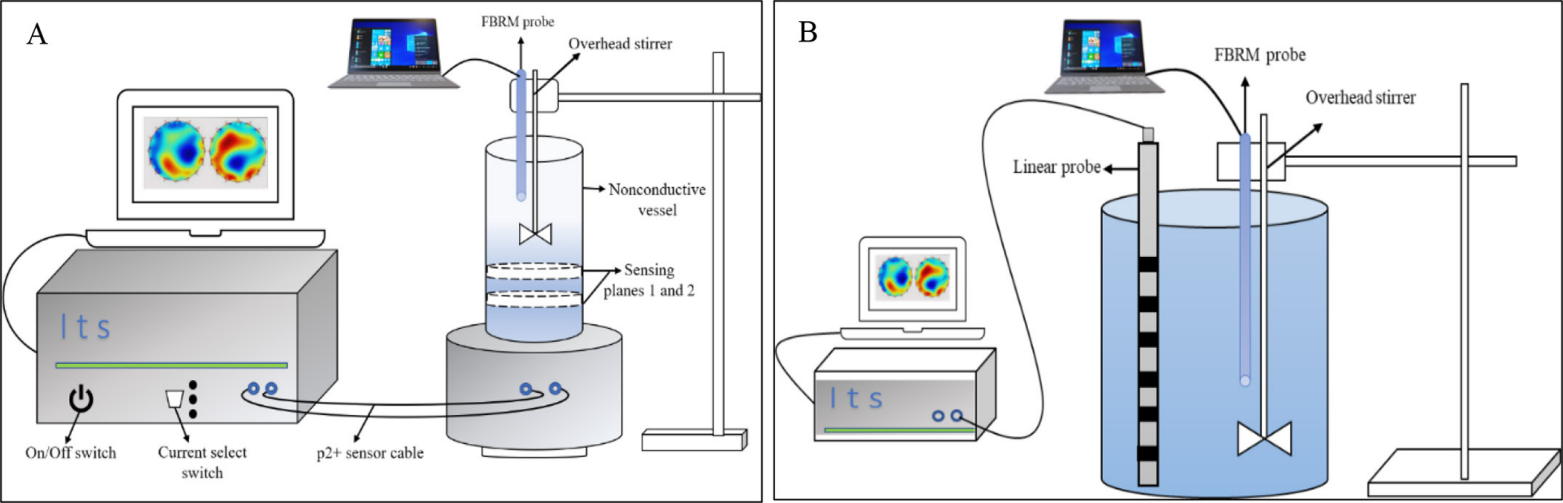
Schematic of the experimental setup for measuring the rehydration of milk protein concentrate powders by electrical resistance tomography using the (A) circular vessel, and (B) linear probe configurations. FBRM = focus beam reflectance measurement (reference method).

The FBRM-based method is a suitable technique for studying the rehydration of MPC powders (Fang et al., 2010). Similar to results of a previous study (Hauser and Amamcharla, 2016b), the FBRM results in the current study suggested that the dissolution characteristics of MPC powder were influenced by the protein content (data not shown). During rehydration of the MPC powders, counts of large particles (150–300 μm) decreased as they disintegrated into fine (<10 μm) particles. From counts of fine particles, we concluded that MPC85 reached an equilibrium state faster rate than MPI90. We also observed that MPC85 had lower counts of large particles than MPI90. The reduction in counts of fine particles in MPI90 powders indicated that solubility decreased as protein content increased. Similar results were obtained in a study by Gandhi et al. (2017), and it was noted that the rate of increase in fine counts was higher for more-soluble MPC powders. Our interpretation of FBRM data matched the overall trends reported by Crowley et al. (2015) and Hauser and Amamcharla (2016b).

According to Hauser and Amamcharla (2016a), the solubility of MPC powders decreases with increasing protein content. In the present study, we expected a reduction in solubility with higher protein content, and we captured the changes in dissolution characteristics using overall mean conductivity measurements and other derived ERT parameters to quantify the changes in the overall mean conductivity curve. Figure 2 shows the overall mean conductivity of the MPC85 and MPI90 powders using the circular and linear probe configurations, respectively. At the end of the 30-min dissolution time, the overall mean conductivity from the circular configuration was 0.196 ± 0.004 mS/cm for MPC85, which was significantly (*P* < 0.05) different from that of MPI90 (0.179 ± 0.029 mS/cm). It is evident from Figure 2 that the proposed method was able to distinguish the changes in dissolution characteristics for MPC powders.

**Figure 2:**
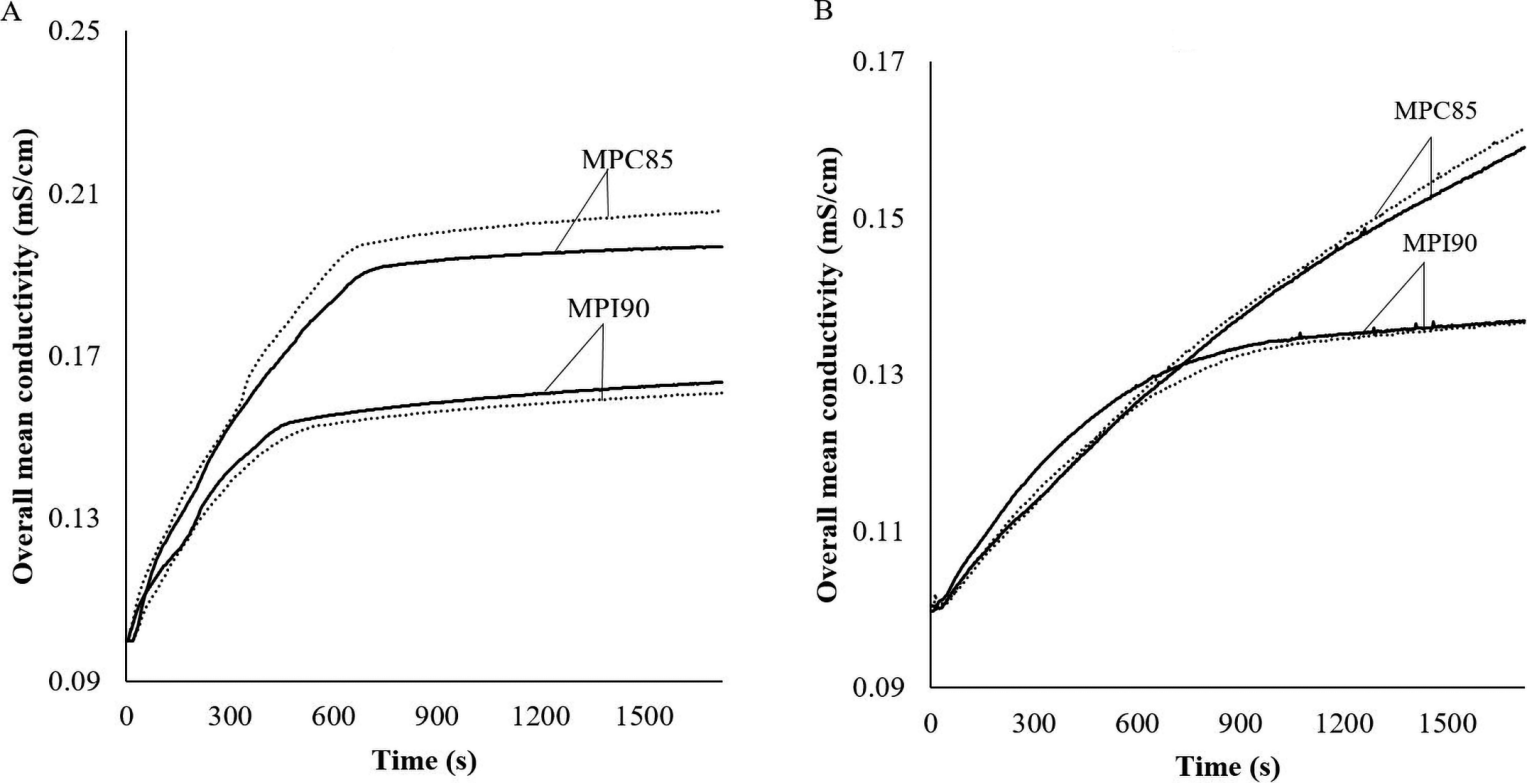
Overall mean conductivity results obtained from the electrical resistance tomography system (A) circular vessel, and (B) linear probe configurations during the dissolution of milk protein concentrate (MPC) powders: MPC85 (milk protein concentration with 85% protein) and MPI90 (milk protein isolate with 90% protein) powders from lot 1 (solid curves) and lot 2 (dotted curves).

Similarly, after the 30-min dissolution time, the overall mean conductivity from the linear configuration was 0.137 ± 0.001 mS/cm for MPC85, which was significantly (*P* < 0.05) different from that of MPI90 (0.160 ± 0.003 mS/cm). Marabi et al. (2008) studied the rehydration characteristics of skim milk powders using conductivity measurements, and they noted that conductivity values increased initially as the powder was rehydrated, likely due to the release of charged species (soluble ions and proteins). An equilibrium conductivity reading was interpreted by the investigators to indicate complete rehydration. Very recently, McSweeney et al. (2021) studied the rehydration profile of MPCs using a conductivity-based method and noted that the release of minerals from powder particles was completed in approximately 3,000 s. They also observed that regular MPC powders exhibited wetting and sinking after approximately 600 s and then reached a plateau.

The conductivity tomogram displayed circular and rectangular conductivity distributions for the circular and linear configurations, respectively (Figure 3). A color scale was used to display the variation in conductivity. Additionally, tomograms of different frames of the same sensor plane or the same frame of different planes can be stacked to form a 3-dimensional image. Figure 3A shows a typical change in tomograms from lot 1 at the end of the 30-min dissolution time for MPC85 and MPI90, using the ERT circular configuration (showing the tomograms at the 2 sensor planes P1 and P2). At the start of the experiment, the geometry was filled with tap water with an average conductivity of 0.1 mS/cm. All tomograms thus showed the blue color. Immediately after the addition of MPC powder, large aggregated powder particles started to disintegrate. The lower conductivity of MPC solution in the bottom layers indicated a lower dissolution rate at the beginning (data not shown). As the dissolution time increased, more minerals were released, and the increase in conductivity was visible. The sensing planes P1 and P2 had an overall mean conductivity of 0.192 mS/cm for MPC85; however, for MPI90, the sensing planes P1 and P2 had an overall mean conductivity of 0.161 mS/cm.

**Figure 3:**
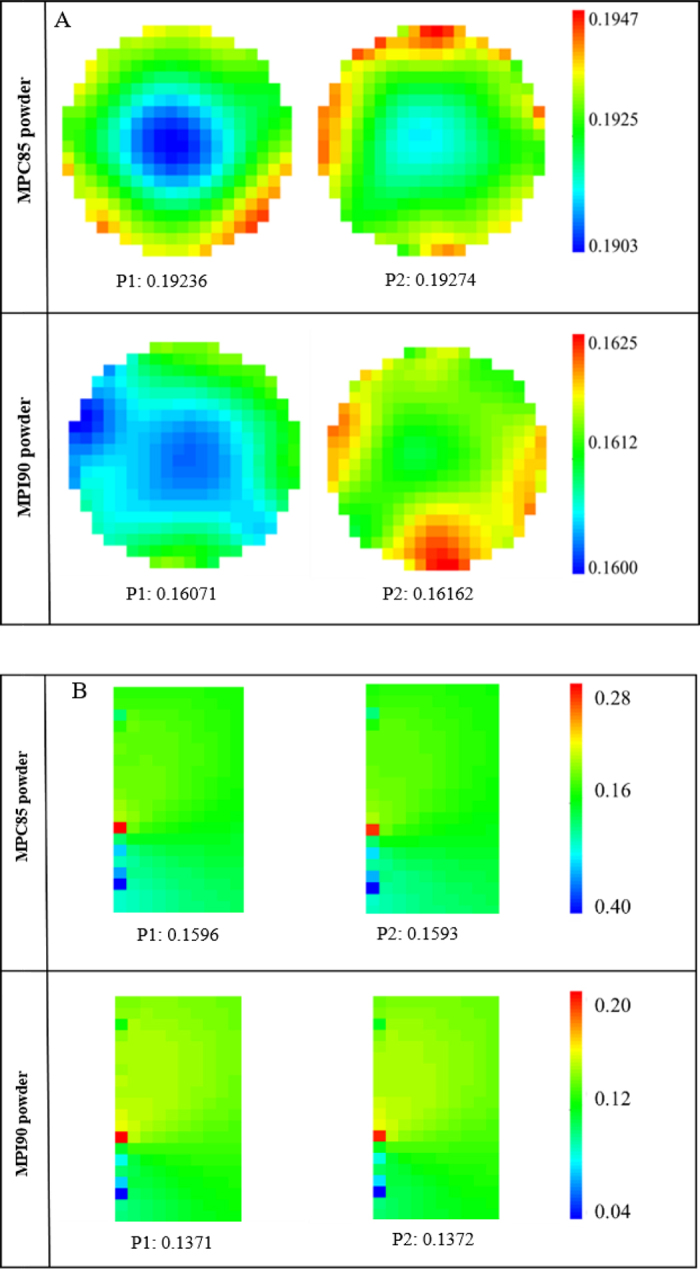
Representative tomogram images obtained from the electrical resistance tomography system (A) circular vessel and (B) linear probe configurations, showing the sensing planes P1 and P2 at the end of the 30-min dissolution time of MPC85 (milk protein concentrate with 85% protein) and MPI90 (milk protein isolate with 90% protein) powders.

Similarly, an extracted tomogram using the linear configuration at the end of dissolution time is shown in Figure 3B. The sensing planes P1 and P2 had an overall mean conductivity of 0.159 mS/cm for MPC85; however, for MPI90, sensing planes P1 and P2 had an overall mean conductivity of 0.137 mS/cm. The ERT tomograms changed remarkably throughout the dissolution time but remained relatively constant after 15 min of dissolution. Thus, ERT enabled real-time insights into patterns of dissolution, and tomograms exhibited pronounced changes that were predominantly related to ions, amino acids (basic and acidic), and proteins, but not to lactose and lipids (Therdthai and Zhou, 2001). Mucchetti et al. (1994) demonstrated that skim milk ultrafiltrate had a similar conductivity to milk, whereas the retentate from diafiltration of skim milk had a significantly lower conductivity. Therefore, it is soluble ions and not the colloidal phase that has the dominant influence on conductivity in milk (Zhuang et al., 1997).

Furthermore, to quantitatively understand the changes in the powder dissolution characteristics in terms of the overall mean conductivity, the relative dissolution index and the slope at a dissolution time of 3 min were derived. The relative dissolution indices for powders in circular and linear configurations were significantly (*P* < 0.05) different, indicating that powder dissolution characteristics could be differentiated using relative dissolution index alone as an indicator. The relative dissolution index values showed approximately 17% (81.54 ± 0.94%) and 5% (94.72 ± 0.71%) reductions for MPI90 with the ERT circular and linear configurations, respectively. This indicated that at lower protein contents, MPC powder readily disintegrates into primary particles, consequently reducing the dissolution time. As the protein content increased, the particle dispersion rate decreased and, consequently, a decrease in mean conductivity and reduction in the area were observed, indicating a decrease in solubility. For the circular sensor configuration, MPC85 and MPI90 showed the maximum conductivity of 0.20 and 0.16 mS/cm, respectively. In contrast, for the linear probe configuration, MPC85 and MPI90 showed maximum conductivity of 0.16 and 0.13 mS/cm, respectively. Previously, McSweeney et al. (2021) observed a maximum conductivity of approximately 0.5 mS/cm after 90 min of dissolution time for regular, agglomerated, and nitrogen-injected MPC powders. For MPC85, the area was around 313 and 231 mS × s/cm for the circular and linear sensor configurations, respectively. However, for MPI90, these values significantly (*P* < 0.05) decreased to 260 and 219 mS × s/cm for the circular and linear sensor configurations, respectively. This indicated that MPI90 had 17 and 5% reductions in area for the circular and linear configurations, respectively. Fang et al. (2010) used FBRM to monitor dissolution of MPC powders and described the initial dissolution rate as the slope of the chord length plot over dissolution time; they suggested that a greater magnitude of slope indicates a faster dissolution rate of the MPC powder. In the current study, MPC85 had a higher slope (at 3 min of dissolution) than MPI90 in both configurations, suggesting a faster dissolution rate. Poor solubility is not due to thermal denaturation; rather, it can be related to a decrease in the water transfer needed for rehydration (Mora-Gutierrez et al., 1995), and the reduction in water transfer can be associated with the micelle structure (Schuck et al., 2007). Measurements using ERT only indicated the rehydration of those constituents that are soluble within the time-course of the analysis and do not address the powders having poorly soluble surface skins of casein micelles that remain until complete rehydration. It was reported by Mimouni et al. (2010) that minerals (nonmicellar material) are freely released during rehydration of MPC powders; however, protein dispersion is the rate-limiting stage.

A fast and robust method using ERT was developed to characterize the dissolution of high-protein dairy powders; FBRM measurements were used as a reference method. From the overall mean conductivity graphs, the relative dissolution index, the area under the conductivity curve, and the slope at 3 min of dissolution time were derived to characterize the rehydration behavior of the MPC powders. The FBRM data revealed that the dissolution rate decreased as the protein content increased. The ERT tomograms, overall mean conductivity plots, and derived parameters clearly showed the potential to characterize powder dissolution using the ERT-based method. As the dissolution time increased, more mineral ions were released from the powder particles, thereby increasing the conductivity. Moreover, ERT provided a 3-dimensional visualization of powder dissolution for monitoring overall dissolution. The findings of this study further emphasize that both ERT configurations were able to capture differences in the dissolution of MPC85 and MPI90 powders. Overall, ERT could be a low-cost, fast, and reliable method to characterize the dissolution behavior of high-protein dairy powders.
